# Prognostic implications of preoperative, postoperative, and dynamic changes of alpha-fetoprotein and des-gamma (γ)-carboxy prothrombin expression pattern for hepatocellular carcinoma after hepatic resection: a multicenter observational study

**DOI:** 10.3389/fonc.2024.1425292

**Published:** 2024-06-05

**Authors:** Shi-Chuan Tang, Ye-Ye Wu, Zhi-Wen Lin, Qing-Jing Chen, Cong Luo, Yun-Tong Li, Jun Fu, Li-Fang Zheng, Peng-Hui You, Song You, Wu-Yi You, Ke-Can Lin, Wei-Ping Zhou, Kong-Ying Lin, Yong-Yi Zeng

**Affiliations:** ^1^ Department of Hepatopancreatobiliary Surgery, The First Clinical Medical College of Fujian Medical University and First Affiliated Hospital of Fujian Medical University, Fuzhou, China; ^2^ Department of Hepatopancreatobiliary Surgery, Mengchao Hepatobiliary Hospital of Fujian Medical University, Fuzhou, China; ^3^ Department of Hepatic Surgery II, Eastern Hepatobiliary Surgery Hospital, Navy Medical University (Second Military Medical University), Shanghai, China; ^4^ Department of Hepatopancreatobiliary Surgery, The People’s Hospital of Zizhong County, Zizhong, China; ^5^ Department of Hepatobiliary Surgery, Zhongshan Hospital of Xiamen University, Xiamen, China; ^6^ Bioinformatics Sample Bank, Biobank in Mengchao Hepatobiliary Hospital of Fujian Medical University, Fuzhou, China; ^7^ Department of Radiation, Mengchao Hepatobiliary Hospital of Fujian Medical University, Fuzhou, China; ^8^ Department of Hepatobiliary Surgery, Eastern Hepatobiliary Surgery Hospital, Navy Medical University (Second Military Medical University), Shanghai, China

**Keywords:** hepatocellular carcinoma, alpha-fetoprotein, des-gamma (γ)-carboxy prothrombin, resection, prognosis

## Abstract

**Background:**

The utility of pre- and post-operative alpha-fetoprotein (AFP) and des-gamma (γ)-carboxy prothrombin (DCP) expression patterns and their dynamic changes as predictors of the outcome of hepatic resection for hepatocellular carcinoma (HCC) has yet to be well elucidated.

**Methods:**

From a multicenter database, AFP and DCP data during the week prior to surgery and the first post-discharge outpatient visit (within 1-2 months after surgery) were collected from patients with HCC who underwent hepatectomy. AFP-DCP expression patterns were categorized according to the number of positive tumor markers (AFP ≥ 20ng/mL, DCP ≥ 40mAU/mL), including double-negative, single-positive, and double-positive. Changes in the AFP-DCP expression patterns were delineated based on variations in the number of positive tumor markers when comparing pre- and post-operative patterns.

**Results:**

Preoperatively, 53 patients (8.3%), 337 patients (52.8%), and 248 patients (38.9%) exhibited double-negative, single-positive, and double-positive AFP-DCP expression patterns, respectively. Postoperatively, 463 patients (72.6%), 130 patients (20.4%), and 45 patients (7.0%) showed double-negative, single-positive, and double-positive AFP-DCP expression patterns, respectively. Survival analysis showed a progressive decrease in recurrence-free (RFS) and overall survival (OS) as the number of postoperative positive tumor markers increased (both *P* < 0.001). Multivariate analysis showed that postoperative AFP-DCP expression pattern, but not preoperative AFP-DCP expression pattern, was an independent risk factor for RFS and OS. Further analysis showed that for patients with positive preoperative markers, prognosis gradually improves as positive markers decrease postoperatively. In particular, when all postoperative markers turned negative, the prognosis was consistent with that of preoperative double-negative patients, regardless of the initial number of positive markers.

**Conclusions:**

AFP-DCP expression patterns, particularly postoperative patterns, serve as vital sources of information for prognostic evaluation following hepatectomy for HCC. Moreover, changes in AFP-DCP expression patterns from pre- to post-operation enable dynamic prognostic risk stratification postoperatively, aiding the development of individualized follow-up strategies.

## Graphical Abstract

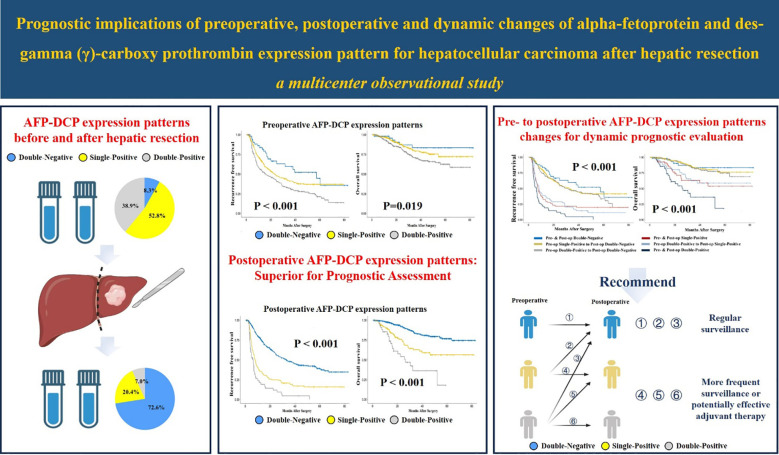

## Introduction

Hepatocellular carcinoma (HCC), responsible for over 85% of primary liver cancer cases worldwide, significantly contributes to global cancer-related mortality (1). Hepatic resection presents potential curative opportunities for HCC patients (2–4). Yet, enhancing long-term prognostic outcomes remains arduous due to the considerable postoperative recurrence rates after hepatectomy (5–7). The inherent heterogeneity of HCC renders the precise prediction of postoperative prognosis challenging, complicating the development of efficacious follow-up protocols and the delineation of personalized therapeutic strategies (8–10).

HCC-specific tumor markers like alpha-fetoprotein (AFP) and des-gamma (γ)-carboxy prothrombin (DCP) offer advantages in their simplicity, speed, and objective, and are broadly utilized in HCC screening, diagnosis, and risk stratification (11–15). AFP and DCP serve as ideal surrogates for the tumor’s biological behavior, with higher levels typically signifying worse malignancy in HCC, irrespective of the tumor’s morphological burden (16, 17). Both the biological behavior and morphological features of HCC hold paramount importance in determining the prognosis for HCC patients. Therefore, many researchers propose incorporating AFP and DCP into the HCC staging system, aiming to compensate for the current system’s focus only on morphological features such as tumor diameter, number, and vascular invasion (18, 19).

However, reliance on the levels of tumor markers at a single ‘time point,’ such as pre-hepatectomy, might not precisely and dynamically evaluate the patient’s prognosis (20, 21). It is crucial to fully utilize ‘time-related variables’ in prognostic predictions, which implies a dynamic evaluation of patient prognosis, especially for patients undergoing hepatectomy with curative intent, where the potential curative effect of hepatectomy should not be disregarded (21, 22). Given the extremely short half-lives of AFP and DCP, 96 and 60 hours, respectively, these markers may serve as ready and effective ‘time-related variables’ for dynamic prognosis prediction in clinical settings (23, 24). Numerous studies have reported that dynamic alterations in AFP levels can effectively indicate the response to various anti-tumor treatments, including hepatectomy (20–22). As post-hepatectomy recurrences frequently arise from micrometastases undetectable by imaging, the dynamic changes in tumor marker levels post-hepatectomy could assist in mirroring the overall condition of the patient, monitoring therapeutic effectiveness, and potentially signaling HCC recurrence earlier than imaging investigations.

However, the efficacy of the combined preoperative and postoperative expression patterns of AFP and DCP and their dynamic changes as predictors of liver resection outcomes has not yet been comprehensively elucidated. In view of this, we collected data from a large cohort of patients who underwent hepatectomy with curative intent for HCC. We focused on preoperative (within 1 week before hepatectomy) and postoperative (within 1–2 months after hepatectomy) levels and related changes in AFP and DCP. Our aim was to assess the prognostic implications of the preoperative, postoperative, and dynamic changes in AFP-DCP expression patterns following hepatic resection for HCC.

## Methods

### Patients selection

In this retrospective study, we analyzed data from patients with newly diagnosed HCC who underwent curative-intent hepatectomy at four medical centers, including the First Affiliated Hospital of Fujian Medical University, Mengchao Hepatobiliary Hospital of Fujian Medical University, Zhongshan Hospital of Xiamen University, and Eastern Hepatobiliary Surgery Hospital of Naval Medical University, from March 2015 to April 2019. Patients with available preoperative (within 1 week before hepatectomy) and first postoperative follow-up (within 1 to 2 months after hepatectomy) serum AFP and DCP data were included. Patients were excluded from the analysis if they: 1) lacked the date on preoperative AFP and DCP within one week before surgery; 2) lacked the date of serum AFP and DCP measurement at the first postoperative follow-up (within 1 to 2 months after hepatectomy); 3) underwent R1 or R2 resection; 4) with recurrent tumor(s) diagnosed by medical imaging at first follow-up after operation; 5) were diagnosed with recurrent HCC or mixed-type HCC; 6) received preoperative anti-tumor therapy; 7) were treated with warfarin; 8) had a history of a second primary malignancy; 9) had incomplete important clinical or follow-up data. This retrospective study was approved by the institutional review boards of each medical center and was conducted according to the ethical guidelines of the 1975 Declaration of Helsinki.

### Clinical variables and definitions

We systematically collected data on patients’ demographic characteristics, operative variables, and clinical-pathologic features. Demographics included gender, age, liver disease etiology, cirrhosis presence, and Child-Pugh classification. Operative variables included type of hepatectomy, extent of hepatectomy, intraoperative blood loss, and transfusion requirement. The types of hepatectomy were divided into anatomical and non-anatomical resections, in line with the 2000 Brisbane definitions for liver anatomy and resections (25). Major hepatectomy was defined as removing three or more of Couinaud’s segments. Clinical-pathological characteristics included laboratory findings and pathologic information. Laboratory findings covered platelet count, total bilirubin, AFP, and DCP. Pathologic information included tumor diameter, number of tumors, degree of tumor differentiation, tumor capsule, satellite nodules, microvascular invasion (MVI), macrovascular invasion, resection margins, and Barcelona Clinic Liver Cancer (BCLC) staging. MVI was defined as tumor cell nests in the vascular lumen lined by endothelial cells under a microscope (26).

### Follow-up and study endpoints

After discharge, patients were regularly reviewed across all centers according to the consistent follow-up scheme. In general, the initial follow-up appraisal was performed 1-2 months post-hepatectomy and comprised routine hematological tests, liver function, and tumor marker assays, along with imaging studies, including lung computed tomography (CT) scans and abdominal ultrasounds or CT/magnetic resonance imaging. This visit’s serum AFP and DCP measurements were recognized as the postoperative levels. Subsequently, patients had follow-ups every 2-3 months for the first two years post-hepatectomy and every 3-6 months after that. If recurrence was suspected, further bone scans or positron emission tomography scans were conducted as needed. Upon recurrence diagnosis, repeat hepatectomy, radiofrequency ablation, transarterial chemoembolization, systemic treatment, or supportive care were adopted, depending on the patient’s tumor status, hepatic functional reserve, and general condition.

The primary endpoints of this study were overall survival (OS) and recurrence-free survival (RFS). OS was defined as the interval between the date of hepatectomy and the date of death or last follow-up. RFS was the interval from the date of hepatectomy to the date of tumor recurrence, death, or last follow-up, whichever occurred first.

### Definition of AFP-DCP expression patterns and the changes

Based on the established standards at each center, the upper normal limits of AFP and DCP in this study were defined as 20 ng/mL and 40 mAU/mL, respectively, and levels exceeding these thresholds were defined as tumor marker positivity. Numerous previous studies have also reinforced these cutoffs for achieving a balance in the sensitivity and specificity of HCC diagnosis. An AFP positivity threshold of 20 ng/mL demonstrated a sensitivity and specificity of 60% and 90%, respectively, for detecting HCC. In comparison, a DCP positivity threshold of 40 mAU/mL yielded sensitivity and specificity of 80% and 90%, respectively (27–29).

AFP-DCP expression patterns were classified based on the number of positive tumor markers, including Double-negative (both AFP and DCP negative), Single-positive (either AFP or DCP positive), and Double-positive (both AFP and DCP positive). Changes in tumor marker expression patterns were defined according to the alterations in preoperative and postoperative tumor marker expression patterns, including preoperative and postoperative both Double-negative group (Pre- & Post-op Double-negative), preoperative Single-positive to postoperative Double-negative group (Pre-op Single-positive to Post-op Double-negative), both preoperative and postoperative Single-positive group (Pre- & Post-op Single-positive), preoperative double-positive to postoperative double-negative group (Pre-op Double-positive to Post-op Double-negative), preoperative Double-positive to postoperative Single-positive group (Pre-op Double-positive to Post-op Single-positive) and preoperative and postoperative both Double-positive group (Pre- & Post-op Double-positive). During the prognostic analysis of changes in AFP-DCP expression patterns, one patient was excluded due to non-conformity with the six changes mentioned above in AFP-DCP expression patterns. This patient manifested a conversion from preoperative AFP single-positivity to postoperative AFP-DCP double-positivity. Notably, the AFP level of the patient escalated from 95.5ng/mL preoperatively to 288.8ng/mL postoperatively, paralleled by an elevation in DCP from 15 mAU/mL preoperatively to 45 mAU/mL postoperatively.

### Statistics

Continuous variables were reported as either mean ± standard deviation or median (interquartile range) and were compared using Student’s t-test or Mann-Whitney U-test. Categorical variables were represented as frequency (percentage) and compared using chi-square or Fisher’s exact tests. The Kaplan-Meier method was deployed to describe postoperative OS and RFS, with intergroup differences compared using the log-rank test. Univariable and multivariable COX proportional hazard regression analyses were performed to identify independent risk factors influencing post-hepatectomy outcomes, with variables achieving P<0.05 in the univariate analysis included in the multivariable Cox regression model. All statistical tests were two-sided, with P<0.05 considered statistically significant. Analyses were conducted using SPSS version 27 (SPSS, Inc., Chicago, IL, USA) and R version 4.1.2 (R Project, Vienna, Austria).

## Results

### Patient characteristics

**Figure 1:**
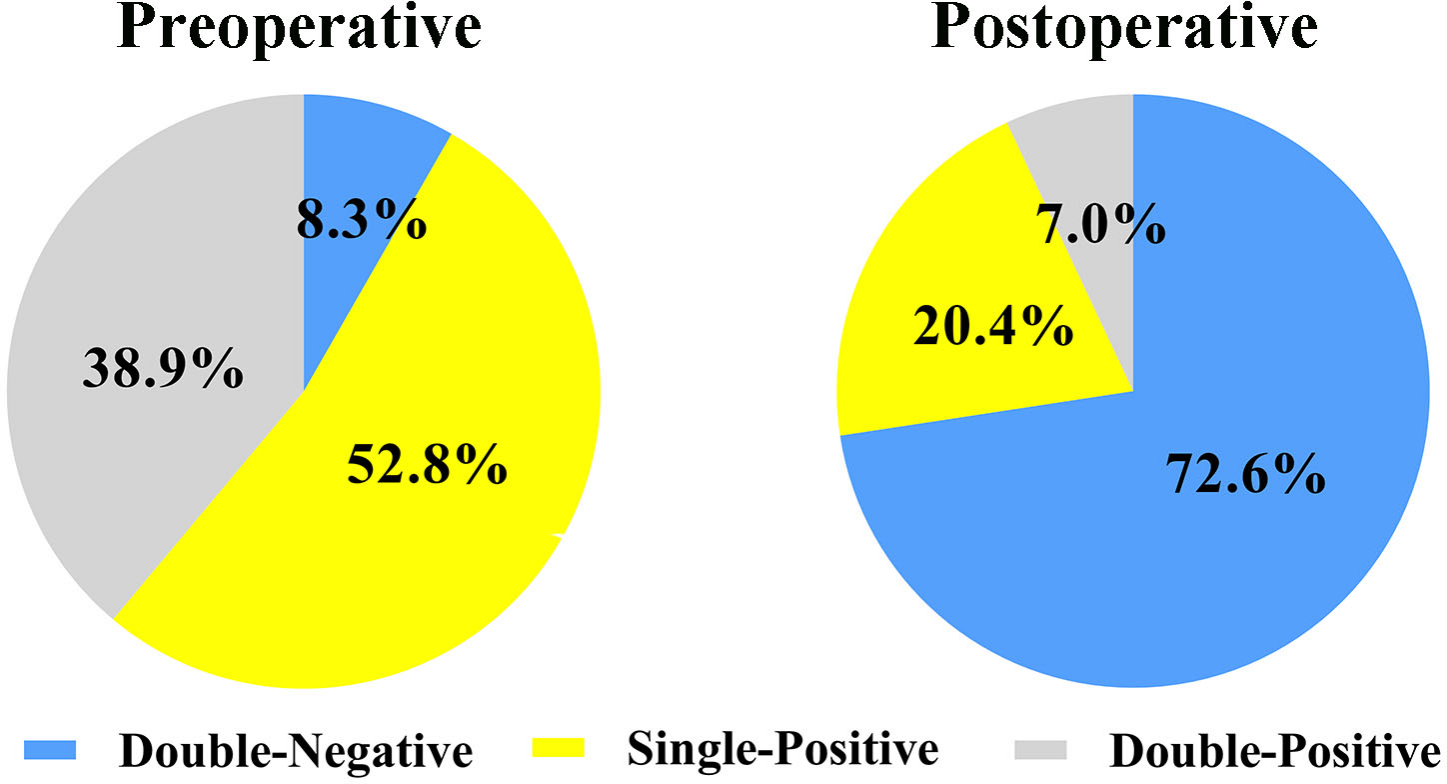
Expression patterns of AFP-DCP before and after hepatectomy. *AFP*, alpha-fetoprotein; *DCP*, des-gamma-carboxy prothrombin; *Double-negative*, both AFP and DCP negative; *Single-positive*, either AFP or DCP positive; *Double-positive*, both AFP and DCP positive.

A total of 638 patients were included in the study, and the baseline clinical characteristics are shown in **Table 1**. Before surgery, 44.3% of patients had positive AFP levels (≥ 20 ng/mL), and 86.0% had positive DCP levels (≥ 40 mAU/mL). Based on the number of positive preoperative tumor markers, tumor marker expression patterns were categorized into three groups: double-negative (53 patients; 8.3%), single-positive (337 patients; 52.8%), and double-positive (248 patients; 38.9%) (**Figure 1**). As shown in **Table 1**, as the number of positive tumor markers increased, the patients exhibited more malignant characteristics, such as larger tumor diameter, poorer tumor cell differentiation, higher incidence of satellite nodules, MVI, and macrovascular invasion, as well as more advanced BCLC staging.

**Table 1:** Comparison of clinicopathological characteristics relative to preoperative tumor marker expression patterns.

Characteristics	Total cohort (n = 638)	Double-negative (N=53)	Single-positive (N=337)	Double-positive (N=248)	*P-value*
**Age**, years	55.6 ± 11.2	56.4 ± 12.1	56.1 ± 10.4	54.7 ± 12.1	0.316
Gender					
Male	557 (87.3)	47 (88.7)	306 (90.8)	204 (82.3)	**0.009**
Female	81 (12.7)	6 (11.3)	31 (9.2)	44 (17.7)	
Etiology					
HBV	563 (88.2)	46 (86.8)	295 (87.5)	222 (89.5)	0.716
HCV	6 (0.9)	1 (1.9)	2 (0.6)	3 (1.2)	
Non-B, non-C	69 (10.8)	6 (11.3)	40 (11.9)	23 (9.3)	
Child-Pugh					
A	547 (85.7)	49 (92.5)	301 (89.3)	197 (79.4)	**0.001**
B	91 (14.3)	4 (7.5)	36 (10.7)	51 (20.6)	
**Cirrhosis**	524 (82.1)	49 (92.5)	271 (80.4)	204 (82.3)	0.104
**PLT**, 10^9^/L	157 ± 78.6	126 ± 64.4	149 ± 69.0	175 ± 89.2	**<0.001**
**Total bilirubin**, μmol/L	17.7 ± 12.4	19.9 ± 12.3	17.0 ± 9.20	18.3 ± 15.7	0.163
**Albumin**, g/L	34.1 ± 8.18	35.1 ± 6.91	34.5 ± 9.85	33.4 ± 5.43	0.081
Tumor number					
Solitary	504 (79.0)	47 (88.7)	270 (80.1)	199 (80.2)	0.320
Multiple	134 (21.0)	6 (11.3)	67 (19.9)	49 (19.8)	
**Tumor diameter**, cm	6.0 ± 3.8	3.1 ± 1.4	5.6 ± 3.5	7.0 ± 4.2	**<0.001**
Tumor differentiation					
I/II	204 (32.0)	28 (52.8)	126 (37.4)	50 (20.2)	**<0.001**
III/IV	434 (68.0)	25 (47.2)	211 (62.6)	198 (79.8)	
Tumor capsule					
Complete	457 (71.6)	40 (75.5)	247 (73.3)	170 (68.5)	0.367
Incomplete	181 (28.4)	13 (24.5)	90 (26.7)	78 (31.5)	
Satellite nodules	148 (23.2)	9 (17.0)	56 (16.6)	83 (33.5)	**<0.001**
**MVI**	346 (54.2)	11 (20.8)	171 (50.7)	164 (66.1)	**<0.001**
**Macrovascular invasion**	83 (13.0)	0 (0)	34 (10.1)	49 (19.8)	**<0.001**
BCLC staging system					
A	447 (70.1)	51 (96.2)	244 (72.4)	152 (61.3)	**<0.001**
B	94 (14.7)	2 (3.8)	57 (16.9)	35 (14.1)	
C	97 (15.2)	0 (0)	36 (10.7)	61 (24.6)	
Hepatectomy					
Anatomical	406 (63.6)	36 (67.9)	223 (66.2)	147 (59.3)	0.183
Non-anatomical	232 (36.4)	17 (32.1)	114 (33.8)	101 (40.7)	
**Intraoperative blood loss**, ml	200 (100, 258)	100 (50, 200)	150 (100, 258)	200 (100, 300)	**<0.001**
**Intraoperative blood transfusion**	97 (15.2)	9 (17.0)	44 (13.1)	44 (17.7)	0.276
Extend of hepatectomy					
Major	142 (22.3)	3 (5.7)	59 (17.5)	80 (32.3)	**<0.001**
Minor	496 (77.7)	50 (94.3)	278 (82.5)	168 (67.7)	
Resection margin					
≥ 1cm	355 (55.6%)	34 (64.2)	197 (58.5)	124 (50.0)	0.0541
< 1cm	283 (44.4%)	19 (35.8)	140 (41.5)	124 (50.0)	

Double-negative, both alpha-fetoprotein (AFP) and des-gamma-carboxy prothrombin (DCP) negative; Single-positive, either AFP or DCP positive; Double-positive, both AFP and DCP positive; HBV, hepatitis B virus; HCV, hepatitis C virus; PLT, platelet; MVI, microvascular invasion; BCLC, Barcelona Clinic Liver Cancer. Bold indicates significant differences between groups (p-value less than 0.05).

Postoperative AFP and DCP expression patterns are shown in **Figure 1**. Patients were categorized into three groups based on the number of postoperative positive tumor markers: 463 patients (72.6%) had double-negative tumor markers, 130 patients (20.4%) had a single-positive tumor marker, and 45 patients (7.0%) had double-positive tumor markers (**Figure 1**). Consistent with the correlation between preoperative tumor marker expression patterns and clinical pathological features, an increase in positive postoperative tumor markers was associated with more advanced tumor characteristics (**Table 2**).

**Table 2:** Comparison of clinicopathological characteristics relative to postoperative tumor marker expression patterns.

Characteristics	Total cohort (n =638)	Double-Negative (N=463)	Single-Positive (N=130)	Double-Positive (N=45)	*P-value*
Age, years	55.6 ± 11.2	56.2 ± 10.8	54.6 ± 12.3	52.7 ± 11.9	0.100
Gender					
Male	557 (87.3)	405 (87.5)	112 (86.2)	40 (88.9)	0.874
Female	81 (12.7)	58 (12.5)	18 (13.8)	5 (11.1)	
Etiology					
HBV	563 (88.2)	403 (87.0)	119 (91.5)	41 (91.1)	0.445
HCV	6 (0.9)	4 (0.9)	1 (0.8)	1 (2.2)	
Non-B, non-C	69 (10.8)	56 (12.1)	10 (7.7)	3 (6.7)	
Child-Pugh					
A	547 (85.7)	406 (87.7)	111 (85.4)	30 (66.7)	**<0.001**
B	91 (14.3)	57 (12.3)	19 (14.6)	15 (33.3)	
Cirrhosis	524 (82.1)	381 (82.3)	102 (78.5)	41 (91.1)	0.159
PLT, 109/L	157 ± 78.6	148 ± 72.2	171 ± 78.0	213 ± 111	**<0.001**
Total bilirubin, μmol/L	17.7 ± 12.4	17.7 ± 13.1	17.6 ± 10.4	18.8 ± 10.8	0.789
Albumin, g/L	34.1 ± 8.18	34.1 ± 8.71	34.6 ± 6.87	32.8 ± 5.56	0.204
Tumor number					
Solitary	516 (80.9)	382 (82.5)	100 (76.9)	34 (75.6)	0.231
Multiple	122 (19.1)	81 (17.5)	30 (23.1)	11 (24.4)	
Tumor diameter, cm	6.0 ± 3.8	5.3 ± 3.6	7.0 ± 3.8	8.8 ± 4.6	**<0.001**
Tumor differentiation					
I/II	204 (32.0)	160 (34.6)	38 (29.2)	6 (13.3)	**0.011**
III/IV	434 (68.0)	303 (65.4)	92 (70.8)	39 (86.7)	
Tumor capsule					
Complete	457 (71.6)	349 (75.4)	90 (69.2)	18 (40.0)	**<0.001**
Incomplete	181 (28.4)	114 (24.6)	40 (30.8)	27 (60.0)	
Satellite nodules	148 (23.2)	79 (17.1)	44 (33.8)	25 (55.6)	**<0.001**
MVI	346 (54.2)	220 (47.5)	89 (68.5)	37 (82.2)	**<0.001**
Macrovascular invasion	83 (13.0)	40 (8.6)	25 (19.2)	18 (40.0)	**<0.001**
BCLC staging system					
A	447 (70.1)	356 (76.9)	73 (56.2)	18 (40.0)	**<0.001**
B	94 (14.7)	49 (10.6)	32 (24.6)	13 (28.9)	
C	97 (15.2)	58 (12.5)	25 (19.2)	14 (31.1)	
Hepatectomy					
Anatomical	406 (63.6)	312 (67.4)	71 (54.6)	23 (51.1)	**0.005**
Non-anatomical	232 (36.4)	151 (32.6)	59 (45.4)	22 (48.9)	
Intraoperative blood loss, ml	200 (100, 258)	200 (100, 258)	200 (100, 258)	258 (200, 400)	**0.034**
Intraoperative blood transfusion	97 (15.2)	68 (14.7)	14 (10.8)	15 (33.3)	**0.001**
Extend of hepatectomy					
Major	142 (22.3)	90 (19.4)	29 (22.3)	23 (51.1)	**<0.001**
Minor	496 (77.7)	373 (80.6)	101 (77.7)	22 (48.9)	
Resection margin					
≥ 1cm	355 (55.6)	268 (57.9)	70 (53.8)	17 (37.8)	**0.031**
< 1cm	283 (44.4)	195 (42.1)	60 (46.2)	28 (62.2)	

Double-negative, both alpha-fetoprotein (AFP) and des-gamma-carboxy prothrombin (DCP) negative; Single-positive, either AFP or DCP positive; Double-positive, both AFP and DCP positive; HBV, hepatitis B virus; HCV, hepatitis C virus; PLT, platelet; MVI, microvascular invasion; BCLC, Barcelona Clinic Liver Cancer. Bold indicates significant differences between groups (p-value less than 0.05).

### Association of preoperative AFP-DCP expression patterns with postoperative outcomes

**Figure 2:**
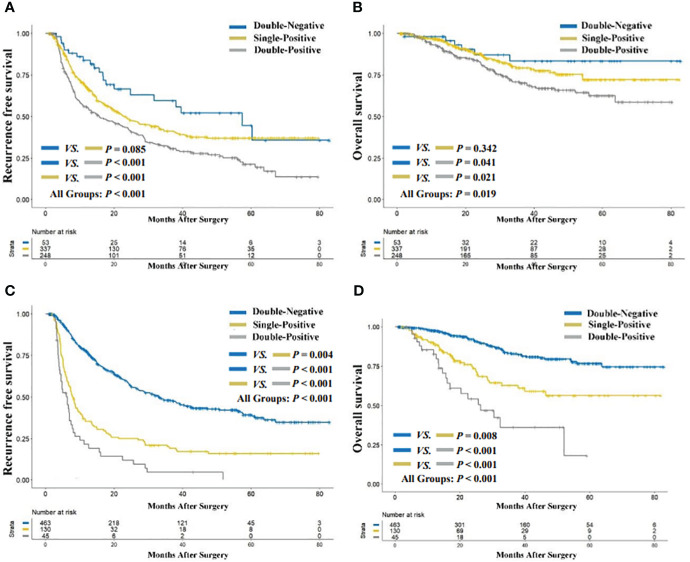
Comparison of recurrence-free **(A)** and overall survival **(B)** among preoperative AFP-DCP expression patterns; comparison of recurrence-free **(C)** and overall survival **(D)** among preoperative AFP-DCP expression patterns. *AFP*, alpha-fetoprotein; *DCP*, des-gamma-carboxy prothrombin.

The median follow-up period for the entire cohort was 42.7 months (range, 2.0 to 82.9). As illustrated in **Figure 2A**, the preoperative double-positive group had a poorer cumulative RFS rate compared to the preoperative double-negative and single-positive groups (double-positive *vs.* double-negative, ***P < 0.001***; double-positive *vs.* single-positive, ***P < 0.001***), while there was no significant difference in the cumulative RFS rate between the preoperative single-positive and double-negative groups (*P = 0.085)*. The cumulative 1-year, 3-year, and 5-year RFS rates for the preoperative double-negative group, preoperative single-positive group, and preoperative double-positive group were 83.8%, 59.5%, and 44.7%; 67.8%, 41.2%, and 37.0%; and 55.7%, 31.8%, and 21.3%. The analysis for OS also showed the same trend across these three groups. The preoperative double-positive group had a poorer cumulative OS rate compared to the preoperative double-negative and single-positive groups *(*double-positive *vs.* double-negative, ***P = 0.041;*** double-positive *vs.* single-positive, ***P = 0.021***), while the preoperative single-positive group’s cumulative OS rate was comparable to that of the preoperative double-negative group (*P = 0.342*) (**Figure 2B**). The cumulative 1-year, 3-year, and 5-year OS rates for the preoperative double-negative group, preoperative single-positive group, and preoperative double-positive group were 98.1%, 83.4%, and 83.4%; 96.3%, 79.3%, and 72.1%; and 92.2%, 70.1%, and 62.6%.

### Association of postoperative AFP-DCP expression patterns with postoperative outcomes

The cumulative RFS rates in patients were shown to decrease progressively with the increasing number of postoperative positive tumor markers (***P < 0.001*** for postoperative double-positive group *vs.* single-positive group; ***P = 0.004*** for postoperative single-positive group *vs.* double-negative group; **Figure 2C**). The cumulative 1-year, 3-year, and 5-year RFS rates for the postoperative double-negative group, postoperative single-positive group, and postoperative double-positive group were 77.5%, 48.2%, and 39.3%; 35.4%, 20.0%, and 16.0%; and 21.4%, 4.7%, and not applicable (NA). Similarly, the OS rate also deteriorated with the increase in the number of postoperative positive tumor markers (***P < 0.001*** for postoperative double-positive group *vs.* single-positive group; ***P = 0.008*** for postoperative single-positive group *vs.* double-negative group; **Figure 2D**). The cumulative 1-year, 3-year, and 5-year OS rates for the postoperative double-negative group, postoperative single-positive group, and postoperative double-positive group were 97.6%, 82.9%, and 76.7%; 89.3%, 62.9%, and 56.4%; and 82.7%, 36.2%, and NA.

### Uni- and multivariable analyses for postoperative outcomes

As per **Table 3**, the multivariable analysis for RFS reveals that the postoperative AFP-DCP expression pattern is the independent poor prognostic factor (double-negative: reference; single-positive: HR 2.178, 95% CI 1.687 - 2.812; double-positive: HR 2.792, 95% CI 1.869 - 4.171; ***P < 0.001***). Other independent risk factors for RFS include HBsAg positivity, poor tumor differentiation, larger tumor diameter, MVI, macrovascular invasion, and resection margins <1cm (**Table 3**). The multivariable analysis for OS demonstrates that the postoperative AFP-DCP expression pattern is an independent risk factor for postoperative OS (double-negative: reference; single-positive: HR 1.629, 95% CI 1.036 - 2.562; double-positive: HR 3.036, 95% CI 1.640 - 5.620; ***P < 0.001***). Other independent risk factors include satellite nodules, MVI, macrovascular invasion, and resection margin < 1cm (**Table 4**).

**Table 3:** Univariable and multivariable Cox regression analyses of risk factors for RFS.

Variables	Univariable Analysis		Multivariable Analysis	
	HR (95% CI)	*P*	HR (95% CI)	*P*
**Age,** years	0.985 (0.976, 0.994)	**0.002**	0.994 (0.984, 1.004)	0.222
**Gender,** male vs female	1.280 (0.928, 1.766)	0.133		
**HBsAg,** positive vs negative	1.642 (1.124, 2.401)	**0.010**	1.667 (1.120, 2.480)	**0.012**
**Child-Pugh,** B vs A	1.594 (1.214, 2.092)	**0.001**	1.184 (0.887, 1.581)	0.251
**Cirrhosis,** present vs absent	0.929 (0.699, 1.236)	0.613		
**PLT,** 10^9^/L	1.001 (1.000, 1.003)	**0.034**	0.999 (0.997, 1.000)	0.051
**Total bilirubin,** μmol/L	1.001 (0.994, 1.008)	0.835		
**Albumin,** g/L	0.988 (0.977, 1.000)	**0.044**	0.997 (0.983, 1.012)	0.711
**Tumor number,** multiple vs solitary	1.427 (1.118, 1.821)	**0.004**	1.144 (0.883, 1.481)	0.309
**Tumor diameter,** cm	1.116 (1.088, 1.145)	**< 0.001**	1.058 (1.022, 1.096)	**0.001**
**Tumor differentiation,** III/IV vs I/II	1.509 (1.199, 1.899)	**< 0.001**	1.321 (1.035, 1.687)	**0.026**
**Tumor capsule,** present vs absent	1.537 (1.226, 1.927)	**< 0.001**	1.233 (0.967, 1.573)	0.091
**Satellite nodules,** presence vs absence	2.230 (1.783, 2.789)	**< 0.001**	1.188 (0.918, 1.538)	0.191
**MVI,** presence vs absence	2.056 (1.655, 2.554)	**< 0.001**	1.560 (1.239, 1.964)	**< 0.001**
**Macrovascular invasion,** presence vs absence	3.088 (2.361, 4.04)	**< 0.001**	1.696 (1.237, 2.324)	**0.001**
**Anatomical resection,** yes vs no	0.660 (0.535, 0.815)	**< 0.001**	0.869 (0.695, 1.088)	0.221
**Extend of hepatectomy,** Major vs Minor	1.224 (0.969, 1.547)	0.090		
**Intraoperative blood loss,** ml	1.001 (1.001, 1.001)	**< 0.001**	1.000 (1.000, 1.001)	0.123
**Intraoperative blood transfusion,** yes vs no	1.213 (0.920, 1.601)	0.172		
**Resection margin,** ≥1cm vs <1cm	0.618 (0.501, 0.761)	**< 0.001**	0.753 (0.601, 0.943)	**0.013**
**Preoperative AFP-DCP expression patterns**				
Single-positive vs Double-negative	1.472 (0.935, 2.316)	0.095	0.902 (0.560, 1.452)	0.671
Double-positive vs Double-negative	2.117 (1.345, 3.331)	**0.001**	0.781 (0.476, 1.281)	0.327
**Postoperative AFP-DCP expression patterns**				
Single-positive vs Double-negative	2.626 (2.079, 3.318)	**< 0.001**	2.178 (1.687, 2.812)	**< 0.001**
Double-positive vs Double-negative	4.877 (3.487, 6.822)	**< 0.001**	2.792 (1.869, 4.171)	**< 0.001**

HBsAg, hepatitis B surface antigen; PLT, platelet; MVI, microvascular invasion; AFP, alpha-fetoprotein; DCP, des-gamma-carboxy prothrombin; Double-negative, both AFP and DCP negative; Single-positive, either AFP or DCP positive; Double-positive, both AFP and DCP positive.

**Bold** indicates statistically significant results in univariable and multivariable analyses.

**Table 4:** Univariable and multivariable Cox regression analyses of risk factors for OS.

Variables	Univariable Analysis		Multivariable Analysis	
	HR (95% CI)	*P*	HR (95% CI)	*P*
**Age,** years	0.988 (0.972, 1.003)	0.122		
**Gender,** male vs female	1.528 (0.842, 2.775)	0.163		
**HBsAg,** positive vs negative	1.547 (0.784, 3.051)	0.208		
**Child-Pugh,** B vs A	1.548 (0.975, 2.457)	0.064		
**Cirrhosis,** present vs absent	1.013 (0.614, 1.671)	0.961		
**PLT,** 10^9^/L	1.002 (1.000, 1.004)	**0.029**	0.998 (0.996, 1.001)	0.169
**Total bilirubin,** μmol/L	1.004 (0.993, 1.015)	0.474		
**Albumin,** g/L	0.990 (0.966, 1.014)	0.404		
**Tumor number,** multiple vs solitary	2.060 (1.406, 3.018)	**< 0.001**	1.297 (0.863, 1.948)	0.211
**Tumor diameter,** cm	1.125 (1.080, 1.171)	**< 0.001**	1.003 (0.948, 1.062)	0.909
**Tumor differentiation,** III/IV vs I/II	1.464 (0.973, 2.204)	0.067		
**Tumor capsule,** present vs absent	1.433 (0.976, 2.105)	0.066		
**Satellite nodules,** presence vs absence	4.036 (2.825, 5.767)	**< 0.001**	1.630 (1.040, 2.554)	**0.033**
**MVI,** presence vs absence	3.206 (2.107, 4.876)	**< 0.001**	2.037 (1.296, 3.204)	**0.002**
**Macrovascular invasion,** presence vs absence	6.977 (4.811, 10.12)	**< 0.001**	4.003 (2.486, 6.446)	**< 0.001**
**Anatomical resection,** yes vs no	0.542 (0.380, 0.775)	**0.001**	0.734 (0.505, 1.068)	0.106
**Extend of hepatectomy,** Major vs Minor	1.843 (1.266, 2.682)	**0.001**	0.797 (0.513, 1.240)	0.315
**Intraoperative blood loss,** ml	1.001 (1.000, 1.001)	**< 0.001**	1.001 (1.000, 1.001)	0.091
**Intraoperative blood transfusion,** yes vs no	1.150 (0.711, 1.860)	0.568		
**Resection margin,** ≥1cm vs <1cm	0.337 (0.232, 0.489)	**< 0.001**	0.551 (0.365, 0.832)	**0.005**
**Preoperative AFP-DCP expression patterns**				
Single-positive vs Double-negative	1.512 (0.647, 3.531)	0.340	0.864 (0.355, 2.102)	0.748
Double-positive vs Double-negative	2.330 (1.010, 5.377)	**0.047**	0.661 (0.265, 1.651)	0.376
**Postoperative AFP-DCP expression patterns**				
Single-positive vs Double-negative	2.737 (1.830, 4.095)	**< 0.001**	1.629 (1.036, 2.562)	**0.035**
Double-positive vs Double-negative	5.920 (3.587, 9.770)	**< 0.001**	3.036 (1.640, 5.620)	**< 0.001**

HBsAg, hepatitis B surface antigen; PLT, platelet; MVI, microvascular invasion; AFP, alpha-fetoprotein; DCP, des-gamma-carboxy prothrombin; Double-negative, both AFP and DCP negative; Single-positive, either AFP or DCP positive; Double-positive, both AFP and DCP positive.

**Bold** indicates statistically significant results in univariable and multivariable analyses.

### Prognostic implications of dynamic changes in AFP-DCP expression patterns

**Figure 3:**
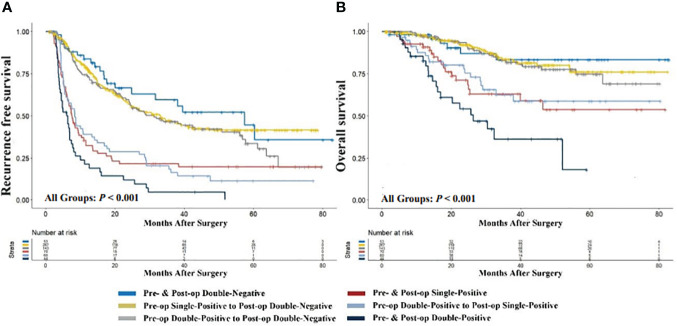
Comparison of recurrence-free **(A)** and overall survival **(B)** with different preoperative to postoperative changes in AFP-DCP expression patterns. *AFP*, alpha-fetoprotein; *DCP*, des-gamma-carboxy prothrombin; *Double-Negative*, both AFP and DCP negative; *Single-Positive*, either AFP or DCP positive; *Double-Positive*, both AFP and DCP positive; *Pre- & Post-op Double-Negative*, the expression pattern of AFP-DCP was double-negative both preoperatively and postoperatively; *Pre-op Single-Positive to Post-op Double-Negative*, the AFP-DCP expression pattern was single-positive preoperatively but converted to double-negative postoperatively; *Pre- & Post-op Single-Positive*, the expression pattern of AFP-DCP was single-positive both preoperatively and postoperatively; *Pre-op Double-Positive to Post-op Double-Negative*, the AFP-DCP expression pattern was double-positive preoperatively but converted to double-negative postoperatively; *Pre-op Double-Positive to Post-op Single-Positive*, the AFP-DCP expression pattern was double-positive preoperatively but converted to single-positive postoperatively; *Pre- & Post-op Double-Positive*, the expression pattern of AFP-DCP was double-positive both preoperatively and postoperatively.

To elucidate the prognostic significance of dynamic changes in AFP-DCP expression patterns, especially in patients with specific AFP-DCP expression patterns, we grouped patients according to different preoperative-postoperative AFP-DCP expression change patterns described in the methodology. As **Figure 3** shows, different change patterns can effectively stratify the prognostic risk for specific patients (***P < 0.001*** for both RFS and OS).

Regardless of whether patients have one or two positive tumor markers preoperatively (presented as single-positive or double-positive), if their postoperative tumor markers are all reduced to negative, their prognosis is similar to patients who are double-negative preoperatively. As shown in **Figure 3**, RFS and OS for the preoperative single-positive to postoperative double-negative group (Pre-op Single-Positive to Post-op Double-Negative group) and the preoperative double-positive to postoperative double-negative group (Pre-op Double-Positive to Post-op Double-Negative) are comparable to the preoperative and postoperative double-negative group (Pre- & Post-op Double-Negative) (all ***P > 0.05***). Furthermore, in patients with positive tumor markers preoperatively, their prognosis progressively improves with the decrease in positive tumor markers postoperatively. In patients with a single-positive tumor marker preoperatively, this prognostic improvement is evident when the preoperative single-positive transitions to the postoperative double-negative group (Pre-op Single-Positive to Post-op Double-Negative), which has a significantly better prognosis than the preoperative and postoperative single-positive group (Pre- & Post-op Single-Positive) (***P < 0.001*** for both OS and RFS). For patients with double-positive preoperatively, the improvement is seen when the preoperative double-positive transitions to the postoperative double-negative group (Pre-op Double-Positive to Post-op Double-Negative), which has better prognosis than the preoperative double-positive transitioning to postoperative single-positive group (Pre-op Double-Positive to Post-op Single-Positive) (***P = 0.004*** for OS, ***P < 0.001*** for RFS), and the latter has better prognosis than the preoperative and postoperative double-positive group (Pre- & Post-op Double-Positive) (***P < 0.001*** for both OS and RFS).

## Discussion

AFP and DCP, widely used in Asian healthcare centers for HCC screening, treatment monitoring, and prognosis evaluation, have been reported for effectiveness (11, 13, 15, 17). Although previous studies have reported correlations of these tumor markers with prognosis following hepatectomy for HCC, scant attention has been directed towards the clinical relevance of these markers’ preoperative and postoperative expression patterns and their corresponding dynamic changes (17–19, 30, 31). In the current study, we explored the significance of the expression patterns of AFP-DCP and the alterations in these patterns post-treatment in forecasting the outcomes of hepatectomy in HCC patients. Our results showed that postoperative, rather than preoperative, expression patterns of AFP-DCP are independent risk factors for RFS and OS. Furthermore, the changes in AFP-DCP expression patterns pre- and post-surgery allow dynamic assessment of the prognosis of patients with a certain preoperative AFP-DCP expression pattern, *i.e.*, patients with positive preoperative tumor markers display progressively improving prognosis with an increasing number of postoperative positive tumor markers turning negative. The findings of this study may aid clinicians in dynamically and accurately identifying high-risk postoperative patients early, thus playing a significant role in enhancing patient counseling and determining the level of immediate postoperative monitoring required.

Previous studies have substantiated the effectiveness of AFP or DCP as prognostic markers for HCC (18, 19, 30, 31). The prognostic relevance of AFP and DCP may be attributed to the hypothesis that their expression levels are not only related to tumor morphological parameters but also to the biological behavior of the tumor, both of which are important factors for patient prognosis (27, 32–35). Furthermore, AFP and DCP levels can be elevated independently in individual patients and are not necessarily correlated, suggesting that AFP and DCP have complementary roles in reflecting the aggressiveness of HCC. For instance, our findings suggest a progressive deterioration of tumor morphological and invasive burden with an increased count of positive tumor markers, and further survival analyses showed the worst cumulative RFS and OS in patients with double-positive tumor markers. This highlights that assessing a single tumor marker might be insufficient to evaluate the prognosis of patients. Thus, a concurrent focus on both AFP and DCP may provide more comprehensive information on tumor biology and prognosis than a singular focus on either marker.

The dynamic evaluation of patients’ prognosis is of paramount importance in prognostic predictions (21, 22, 36, 37). Since the half-lives of AFP and DCP are approximately 4 and 2.5 days, respectively, post-treatment levels of tumor markers obtained at the first postoperative follow-up visit of patients in our study (within 1-2 months post-hepatectomy) were largely unaffected by baseline levels of tumor markers before treatment (23). Our multivariate analysis showed that the postoperative AFP-DCP expression pattern, but not the preoperative AFP-DCP expression pattern, was an independent risk factor for RFS and OS. This underscores the potential advantage of dynamically assessing patient prognosis based on postoperative AFP-DCP expression patterns over solely relying on preoperative patterns, especially for patients undergoing hepatectomy, where the potential efficacy of hepatectomy cannot be ignored. Therefore, clinicians need to pay more attention to the postoperative AFP-DCP expression pattern after liver resection in patients with HCC in clinical practice.

An interesting finding of this study is that the dynamic assessment of prognosis in a specific population can be achieved by integrating changes in AFP-DCP expression patterns pre- and post-operatively. As indicated by our research, the prognosis of patients whose tumor markers turn negative after hepatectomy significantly improves, and the prognosis further ameliorates with a decrease in the number of positive tumor markers.

Specifically, for patients with a single positive tumor marker preoperatively, if the tumor marker drops to negative levels postoperatively, their prognosis is significantly better than those with continuing positive markers postoperatively, and is nearly equivalent to patients with negative tumor markers preoperatively (**Figure 3**). Similarly, for patients positive for both AFP and DCP preoperatively, the prognosis progressively improves as the number of positive tumor markers turning negative postoperatively increases (**Figure 3**), particularly for those reaching double negative postoperatively, whose prognosis is akin to preoperatively double-negative patients. Thus, monitoring changes in AFP-DCP expression patterns can help in the precise and dynamic assessment of patient prognosis, which allows surgeons to better counsel patients and aid in devising follow-up schedules and adjunctive treatment plans to optimize postoperative outcomes.

In interpreting the results of this study, certain limitations should be considered. Firstly, this research is a retrospective cohort study, where biases may be unavoidable. Secondly, the study was conducted in an area with a high prevalence of the Hepatitis B Virus. Thirdly, this study did not include other clinically reported HCC tumor markers, such as Lens culinaris agglutinin-reactive fraction of AFP, due to it not being routinely tested across all centers. Fourth, only patients who had open hepatectomy were included in this study; patients undergoing laparoscopic hepatectomy need additional validation. Lastly and most importantly, the cut-off values for AFP and DCP used in this study are based on each center’s standards. Although these cutoff values are supported by many previous studies, it’s undeniable that the impact of hepatectomy itself could lead to a mild elevation in tumor markers, causing false positivity. For example, 4 cases (0.6%) in our study showed a postoperative increase in tumor markers, three of which were positive for DCP preoperatively, with postoperative DCP levels dropping to negative but AFP levels rising to positive (rising from 11.6 ng/mL to 23.8 ng/mL, 12ng/mL to 32.5 ng/mL, and 11 ng/mL to 39 ng/mL respectively). One case was positive for AFP preoperatively and became positive for both AFP and DCP postoperatively, with AFP rising from 95.5ng/mL preoperatively to 288.8 ng/mL, and DCP increasing from 15 mAU/mL to 45 mAU/mL. Therefore, future studies with larger sample sizes are needed to determine the optimal cut-off values for AFP and DCP.

In conclusion, the expression patterns of AFP and DCP, particularly postoperative expression patterns, serve as vital sources of information for prognostic evaluation following hepatectomy for HCC. Moreover, combining preoperative and postoperative changes in the expression patterns of these two biomarkers can enhance dynamic postoperative prognostic risk stratification, aiding in the establishment of personalized follow-up decision-making.

## Data availability statement

The raw data supporting the conclusions of this article will be made available by the authors, without undue reservation.

## Ethics statement

The studies involving humans were approved by Institutional Review Boards of Mengchao Hepatobiliary Hospital of Fujian Medical University. The studies were conducted in accordance with the local legislation and institutional requirements. The ethics committee/institutional review board waived the requirement of written informed consent for participation from the participants or the participants’ legal guardians/next of kin because All informed consents were waived by the ethics given the retrospective nature of this study.

## Author contributions

S-CT: Writing – review & editing, Writing – original draft, Supervision, Software, Methodology, Investigation, Formal analysis, Data curation, Conceptualization. Y-YW: Writing – original draft, Data curation, Conceptualization. Z-WL: Writing – original draft, Software, Methodology, Data curation. Q-JC: Writing – original draft, Methodology, Investigation, Data curation. CL: Writing – original draft, Project administration, Formal analysis, Data curation. Y-TL: Writing – original draft, Resources, Project administration, Data curation. JF: Writing – original draft, Software, Formal analysis, Data curation. L-FZ: Writing – original draft, Data curation. P-HY: Writing – original draft, Data curation. SY: Writing – original draft, Data curation. W-YY: Writing – original draft, Data curation. K-CL: Writing – review & editing, Project administration, Funding acquisition, Data curation, Conceptualization. W-PZ: Writing – review & editing, Supervision, Resources, Project administration. K-YL: Writing – review & editing, Writing – original draft, Data curation, Conceptualization. Y-YZ: Writing – review & editing, Visualization, Validation, Resources, Project administration, Funding acquisition, Conceptualization.
